# Supplementary material for: A clinically annotated post-mortem approach to study multi-organ somatic mutational clonality in normal tissues
Source: Sci Rep. 2022 Jun 20;12:10322. doi: 10.1038/s41598-022-14240-8 (PMC9209481; doi:10.1038/s41598-022-14240-8)
Supplement: Supplementary file 1 — Supplementary Information 1. [file 41598_2022_14240_MOESM1_ESM.pdf]

Supplementary figures and tables

Fig. S1 Detecting mutational clonality in post-mortem epithelial tissues derived from whole-body donors.

(a) A 4-step methodology to detect mutational clonality. **(b)** DNA integrity gel electrophoresis image for DNA extracted from epidermal eyelid samples from 10 different subjects (indicated by circles) and with a broad range of PMIs. Donors from which samples were sequenced in this study (PM01, PM02) are indicated and labelled in red. The slightly better DNA integrity of samples obtained from these 2 subjects is likely related to the fact that these samples were obtained from fresh tissues, while the other 8 samples were obtained from frozen tissues. Complete gel image shown on the right. (c) Histogram showing the distribution of variant allele frequencies of the 920 somatic mutations that were identified in this study.

Fig. S2 Overview of all identified somatic mutations in this study.

Somatic mutations were called from deep (1000x) targeted sequencing data using Shearwater ML. Heatmap shows all mutated genes with indication of the number of identified mutations per sample. Genes are stratified according to their type (driver genes, housekeeping genes and immune genes as indicated by the grey bar on the right) and ranked from top to bottom by mutation frequency.

Fig. S3 Positive selection signals in driver mutations in oral and skin epithelial tissues.

(a) Barplots showing dN/dS (upper panel) or dNons/dS (lower panel) for 7 genes as indicated. **(b)** Plots showing expected (violin plot, based on simulations) and observed (scatter plots) PP2 values for 7 genes as indicated. Median values indicated by horizontal lines. For both panels, the results from this study are compared with results obtained from another study on healthy skin *(Martincorena et al. 2015*, targeted sequencing 74 genes) and with squamous cell skin cancer (SCC, whole exome sequencing) data as indicated. *, (unadjusted) $P < 0.05$; **, $P < 0.01$; ***, $P < 0.001$; NS, non-significant.

Fig. S4 Clonal alterations in skin and oral epithelia.

Visualization of somatic mutation-driven clones in 0.25 cm^2 healthy skin/oral epithelium for both study donors as indicated. Clones driven by genes for which positive selection signals were found in both skin and oral epithelia are coloured as indicated in bottom legend. Other clones are uncoloured. Clones were positioned randomly with clone sizes and frequency based on somatic mutation data.

Table S1 Main mutation data used in this study.

Main data that underly the results reported in this manuscript, as indicated by sheet names: gene set that was used for the development of the targeted gene panel, with indication of gene type (driver, immune or housekeeping gene), reference and classification in oncogene or tumor suppressor gene (TSG) as derived from COSMIC's Cancer Gene Census v91; mutation annotation format (maf) file; SimSen sequencing results; positive selection analysis in skin/oral epithelium with indication of dN/dS, (median) PP2 and related values.

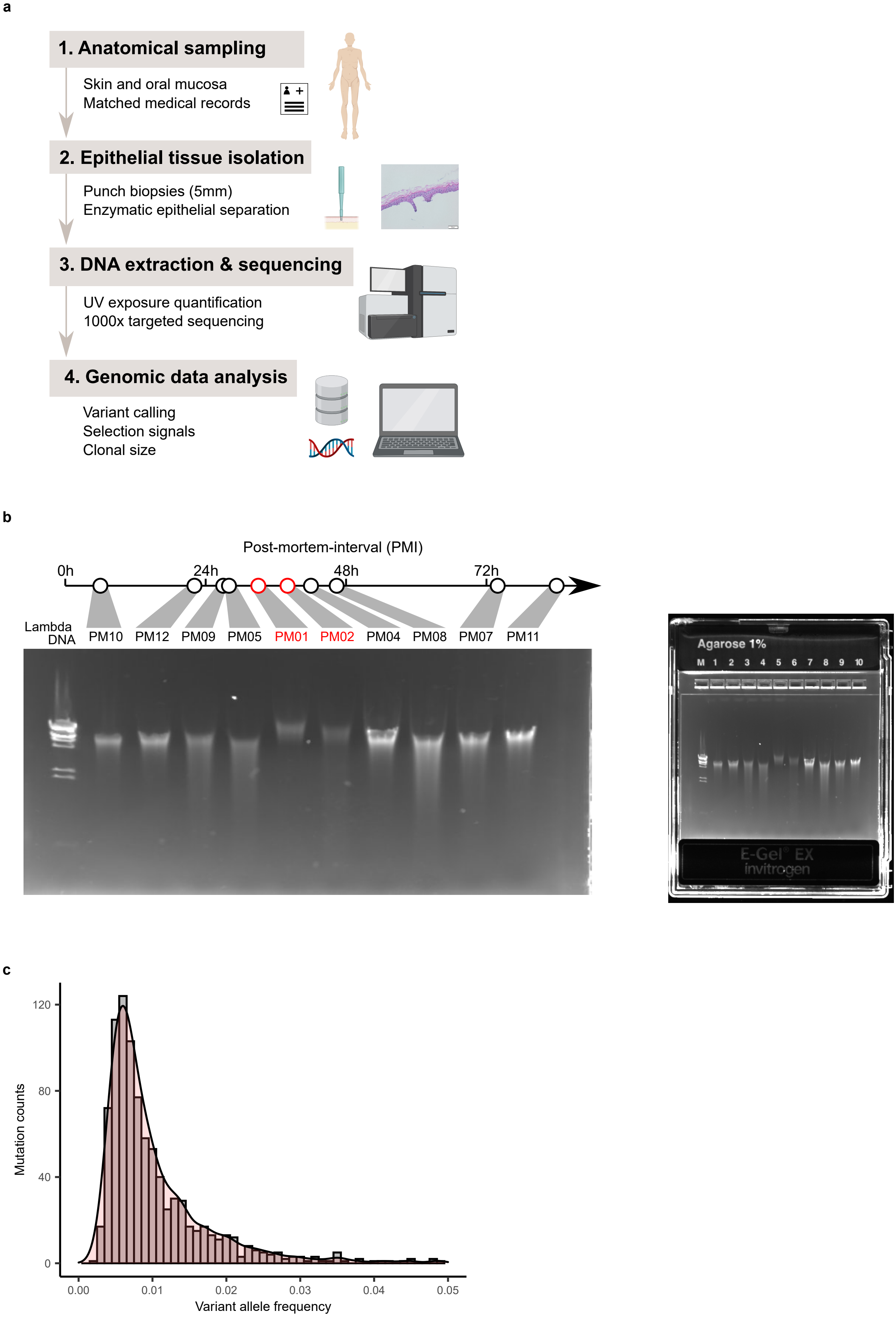

Suppl. figure 1

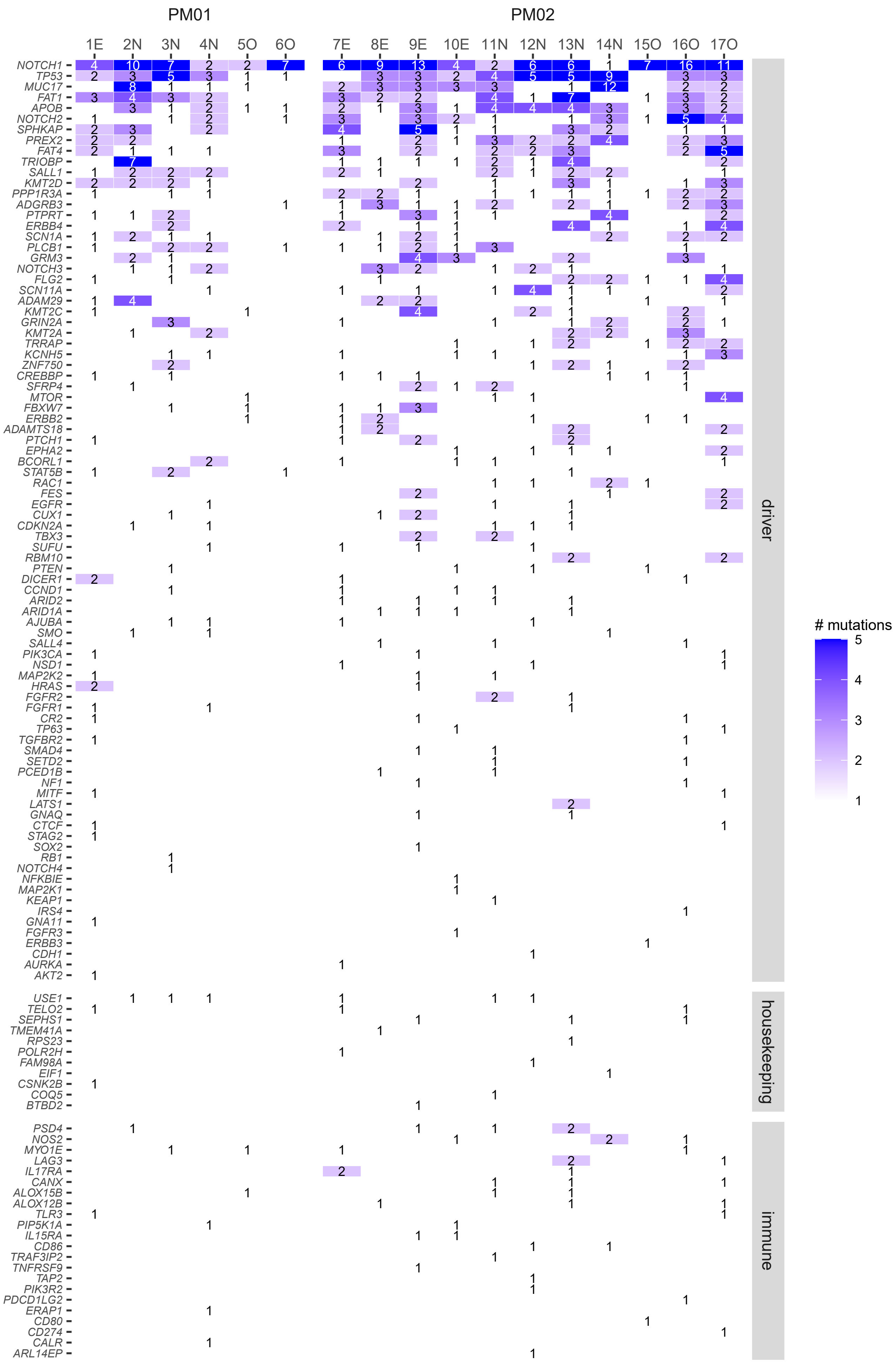

Suppl. figure 2

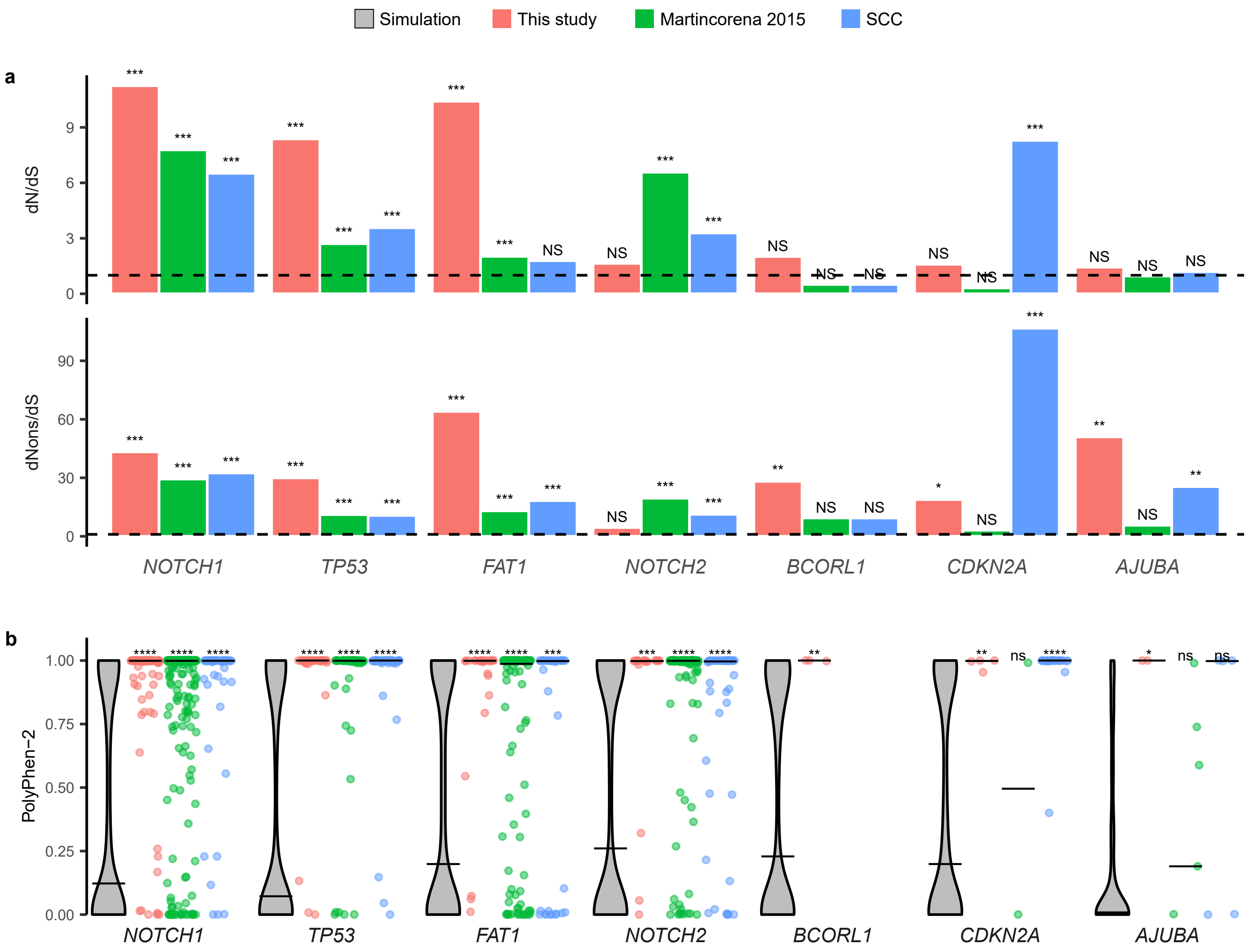

Suppl. figure 3

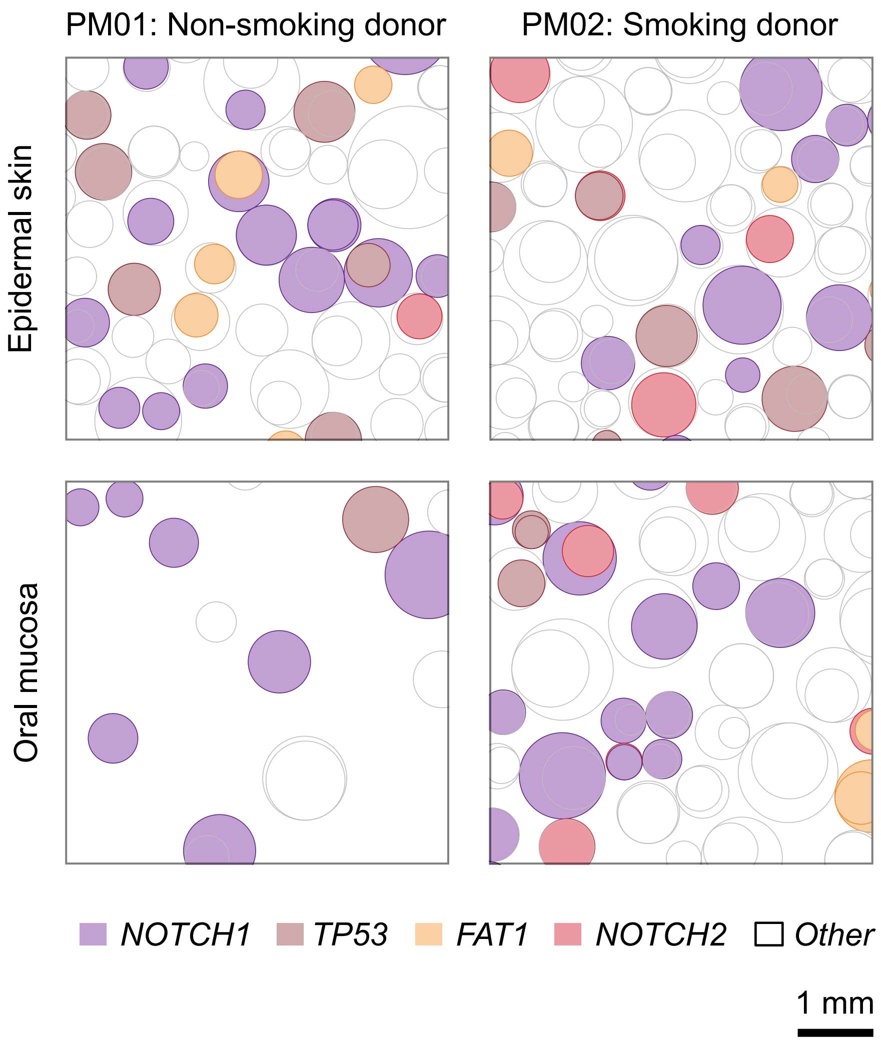

Suppl. figure 4
